# Supplementary material for: Osteoporosis Pre-Screening Using Ensemble Machine Learning in Postmenopausal Korean Women
Source: Healthcare (Basel). 2022 Jun 14;10(6):1107. doi: 10.3390/healthcare10061107 (PMC9222287; doi:10.3390/healthcare10061107)
Supplement: Supplementary file 1 [file healthcare-10-01107-s001.zip › healthcare-1745233-supplementary.pdf]

Supplementary Materials

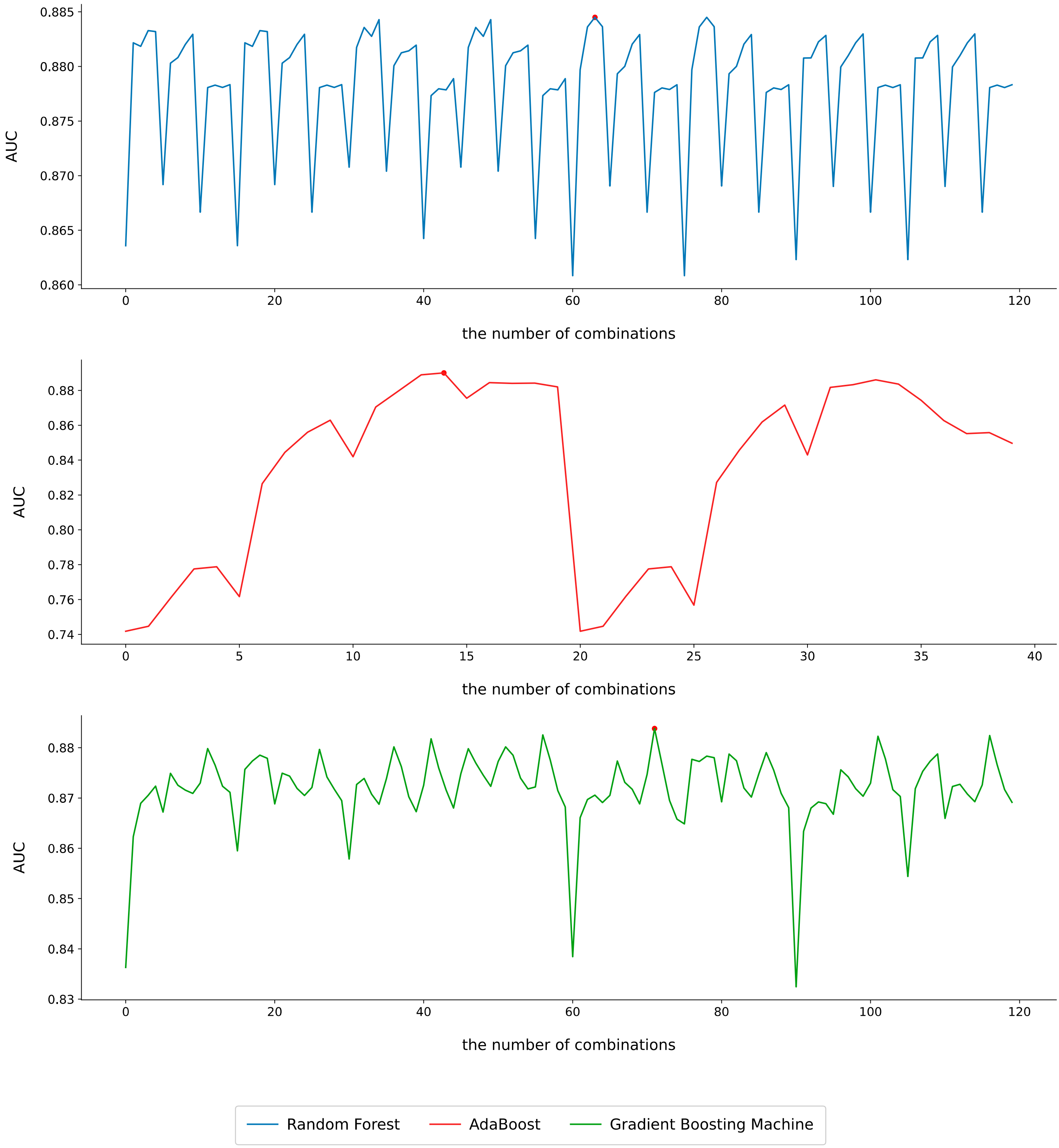

Figure S1. Machine Learning Curve for each best models using Grid search method; Random Forest, AdaBoost and Gradient Boosting Machine. A red dot indicates the combinations of hyperparameters that result in the best performance of each model.

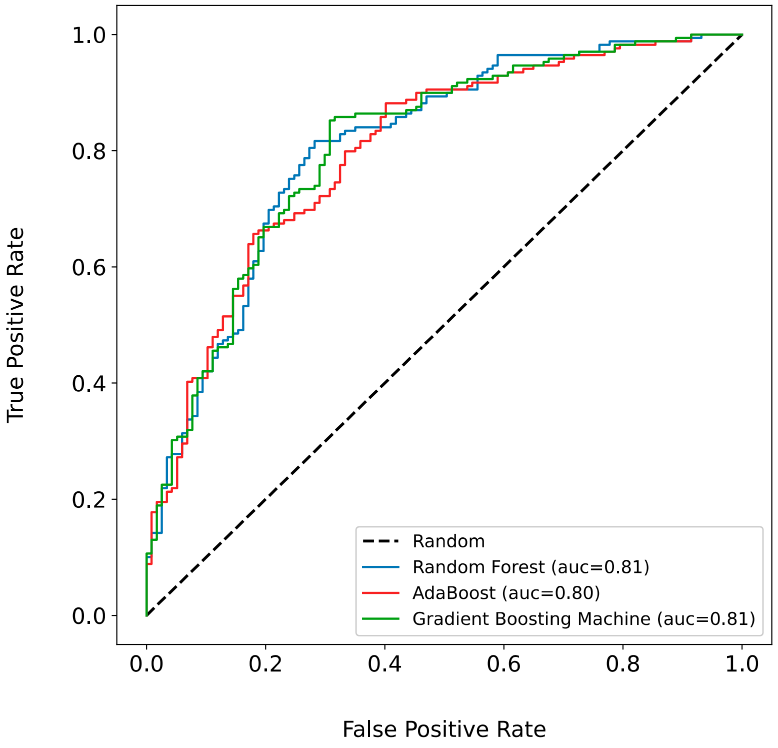

Figure S2. ROC (Receiver Operating Characteristic) Curve for three different best models (Random Forest, AdaBoost and Gradient Boosting Machine) based on 10 selected Checkup features.

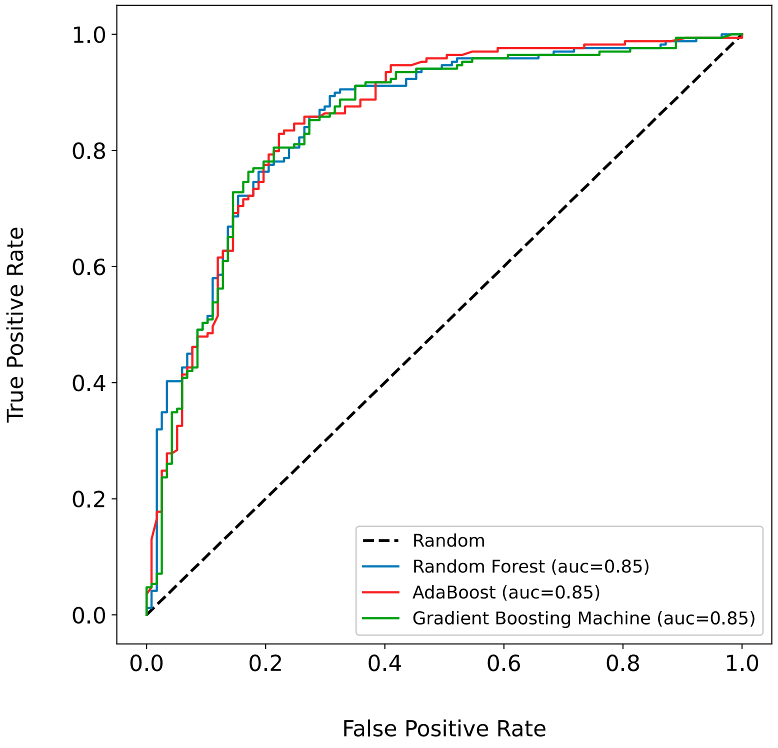

Figure S3. ROC (Receiver Operating Characteristic) Curve for three different best models (Random Forest, AdaBoost and Gradient Boosting Machine) based on 10 selected Survey features.

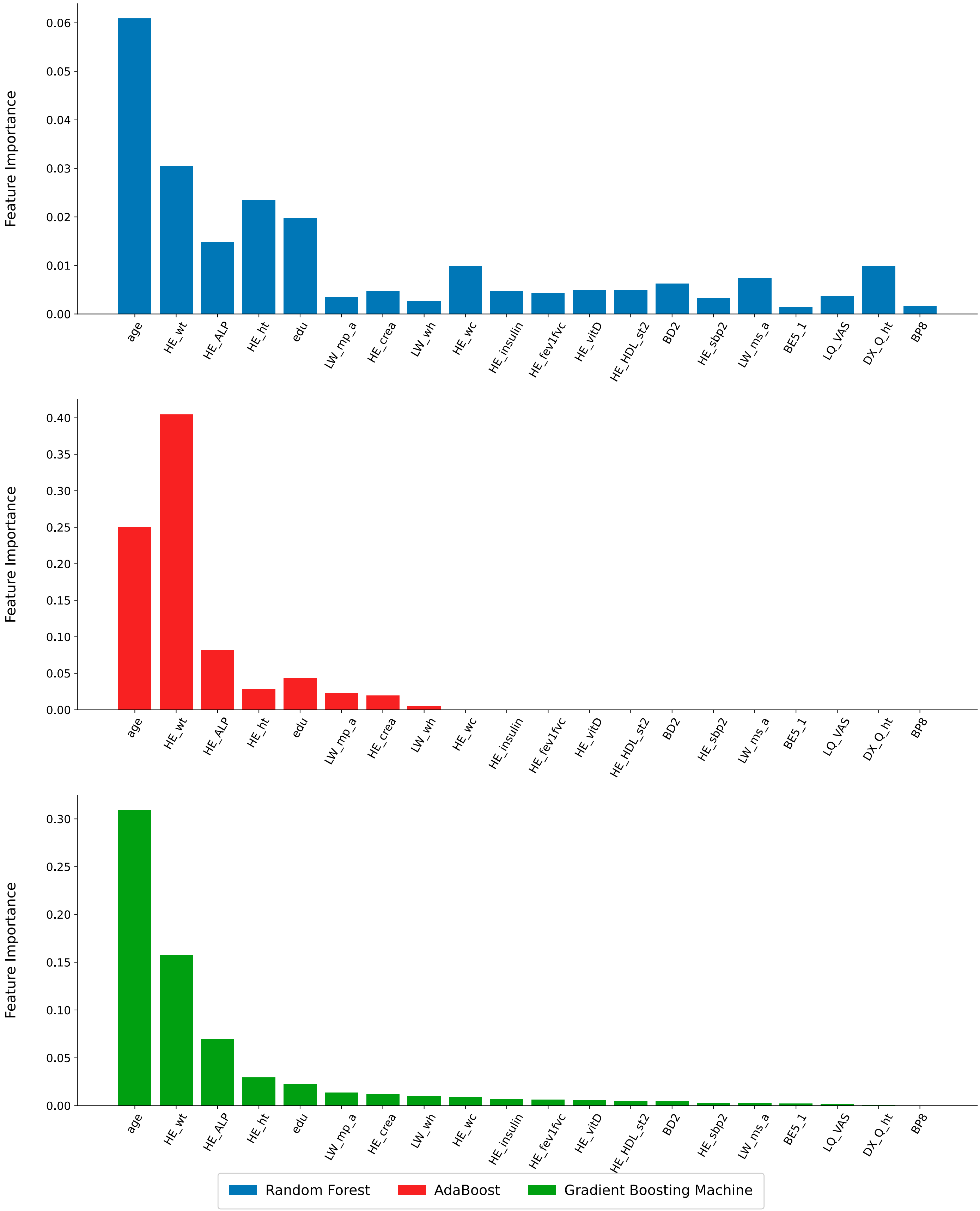

Figure S4. Feature importance bar graph for 20 features measured by three different ensemble machine learning models (Random Forest, AdaBoost and Gradient Boosting Machine).

Table S1. The number of normal and osteoporosis patients distributed in women

Age range	**0 ~ 39**	**40 ~ 49**	**50 ~ 59**	**60 ~ 69**	**70 ~ 79**	**Over 80**
Normal	7	84	391	135	27	1
Osteoporosis	0	11	222	588	697	232

Table S2. Summary of the Model C's Performances on the test set (N =287).

**Model**	**Accuracy**	**Precision**	**Recall**	**AUC**
Random Forest	0.832	0.833	0.893	0.919
AdaBoost	0.849	0.849	0.905	0.921
Gradient Boosting	0.829	0.844	0.869	0.908

* Top performing model is underlined. AUC: Area Under the Curve. GBM: Gradient Boosting Machine
